# Trends in Intermittent Scanning Continuous Glucose Monitoring Usage in The Netherlands—An Opportunity for Elderly Individuals with Diabetes

**DOI:** 10.3390/jcm13237301

**Published:** 2024-11-30

**Authors:** Riemer A. Been, Rijk O. B. Gans, Pratik Choudhary, André P. van Beek, Peter R. van Dijk

**Affiliations:** 1 Department of Endocrinology, University Medical Center Groningen, University of Groningen, 9713 GZ Groningen, The Netherlands; a.p.van.beek@umcg.nl (A.P.v.B.); p.r.van.dijk@isala.nl (P.R.v.D.); 2 Department of Internal Medicine, University Medical Center Groningen, University of Groningen, 9713 GZ Groningen, The Netherlands; 3 Department of Internal Medicine, Curaçao Medical Center, J. H. J. Hamelbergweg, Willemstad, Curacao; 4 Leicester Diabetes Centre, Leicester General Hospital, University of Leicester, Leicester LE5 4PW, UK; pratik.choudhary@leicester.ac.uk; 5 Department of Internal Medicine, Diabetes Center, Isala Zwolle, 8025 AB Zwolle, The Netherlands

**Keywords:** flash glucose monitoring, blood glucose self-monitoring, diabetes

## Abstract

**Background:** Intermittent scanning continuous glucose monitoring (is-CGM) technology has gained widespread adoption and is known to improve glycemic control and quality of life for persons with diabetes. The elderly may lag behind in their adoption of the technology, which could be a potential avenue for improving quality of care. In this study, we investigated the adoption of is-CGM technology in the Dutch population, including effects of age. **Methods:** A retrospective observational study was performed using data from the Drug Information Project, a public database hosted by the Dutch National Health Care Institute. The database contained information concerning healthcare reimbursements from 2017 until 2022 and covered approximately 95% of the total population. Data concerning is-CGM and fast-acting insulin reimbursements were extracted, identifying actual and potential is-CGM users, who were subdivided into the categories 0–24, 25–44, 45–64, 65–74 and ≥75 years old. **Results:** From 2017 until 2022, is-CGM usage rapidly increased: from 38 to 82.050 actual users. The age categories 0–24 and 25–55 showed the highest is-CGM usage (62% and 84% of the potential population in 2022, respectively), and 65–74 and ≥75 the least (38% and 33%, respectively). However, the elderly had higher growth rates (+75% in the category ≥75 from 2021 to 2022) compared to the youngest (+54% in the category 0–24 in the same period). **Conclusions:** Data from this study demonstrate that the elderly lag behind in is-CGM adoption. Given the potential advantages of is-CGM for elderly persons with diabetes, we argue that strategies should be developed to address this (paradoxical) underutilization of is-CGM.

## 1. Introduction

Usage of Freestyle Libre intermittent scanning continuous glucose monitoring (is-CGM) technology improves the glycemic control and quality of life of people with both type 1 and type 2 diabetes (DM) [1,2,3]. This, combined with their ease of use, has been instrumental to the widespread adoption of is-CGM.

In November 2017, it was decided to reimburse is-CGM technology in the Netherlands for children with type 1 diabetes (T1DM); adults with T1DM and either severe hypoglycemic episodes, hypoglycemia unawareness or severe dysglycemia (HbA1c > 8.0% [64 mmol/mol]); and women with diabetes of any type either currently pregnant or planning to be so [4]. After December 2019, is-CGM reimbursement criteria were expanded to cover all Dutch persons with DM that use intensive treatment (e.g., >2 injections of insulin, with one of these injections being short-acting insulin, per day).

However, while is-CGM has been popular and adopted by many persons with DM, the popularity has not spread equally to all subpopulations of people with diabetes [5]. One such population that lags behind in technology adaptation has been the elderly. Older people are often assumed, whether warranted or not, to be less suitable for the use of innovative technologies, possibly due to visual and hearing impairments, decreased dexterity, and possible data overload, amongst other factors [6]. However, there have been studies that do show benefits of this technology in this population similar to other populations, including a decreased number of hypoglycemic episodes [7] (especially relevant due to increased fall risk in this population [8]) and improved quality of life for users themselves, as well as their partners [9]. Moreover, it has been shown that, even among those who already measure their glucose levels frequently, CGM devices can still cause the same improvements in glycemic control and hypoglycemia prevention [10]. This strongly suggests that is-CGM is feasible in the elderly population, and as such, might form a potential avenue to improve quality of care and diabetes outcomes.

We have previously described nationwide data from the Dutch population that clearly demonstrated that the elderly lag behind in the use of is-CGM. From 2019 to 2021, those aged 65 to 74 were consistently prescribed is-CGM technology less often than the national average (2.7% vs. 7.4%, 22.7% vs. 27.6%, and 28.3% vs. 31.5% of the eligible populations for 2019, 2020, and 2021, respectively), with even lower prescription rates for those aged 75 and older (1.1%, 12.2% and 19.0% for 2019, 2020 and 2021, respectively) [11].

Considering the potential benefits of is-CGM in the elderly and our previous observations, it remains important to monitor the adoption of is-CGM so as to identify potential areas of improvement of care. To that end, we investigated the trends in is-CGM usage over the past few years across different age groups among the Dutch population with insulin-dependent diabetes.

## 2. Materials and Methods

This is a descriptive retrospective observational study using data from the Drug Information Project (‘Genees- en hulpmiddelen Informatie Project’), a publicly available database hosted by the Dutch National Health Care Institute [12]. This database contains information concerning the Dutch healthcare reimbursements part of the statutory national healthcare reimbursement scheme. This covers approximately 95% of the total Dutch population. Data concerning reimbursements of medications and the glucose measurement aids are coded via the usage of Anatomical Therapeutic Chemical (ATC) codes. The is-CGM device reimbursements are coded as ‘F3505’, and fast-acting insulins as ‘A10AB’ [13]. The prescription of fast-acting insulins allows for the identification of the number of potential recipients of reimbursements for is-CGM devices, as the current mandates from the government allow all those with diabetes dependent on intensive insulin treatment (defined as using a basal-bolus scheme, which necessitates the prescription of fast-acting insulins) to use the devices, regardless of diabetes type [14]. The prescription of is-CGM devices then allows for the identification of those that receive the reimbursement and thus use the device. Both codes allow for the division of the population into subcategories by registered gender (male or female) and age (the predefined categories 0–4 years old, 5–14, 15–24, 25–44, 45–64, 65–74 and ≥75 for insulin prescriptions, 0–24, 25–44, 45–64, 75–84 and ≥85 for is-CGM prescriptions). Usage of these numbers then allows for the determination of how many of those eligible for reimbursement actually use the device, subdivided by the aforementioned age categories. To allow for proper comparison, the age categories were subdivided into the categories 0–24, 25–44, 45–64 and ≥75 years old. There were no data available to allow for any further differentiation between the groups. Furthermore, at the time of writing (2024), these data were available from 2017, 2018, 2019, 2020, 2021, and 2022, which allowed for the calculation of growth rates between those years. For the variables regarding the potential and actual users, population counts will be given of the total population and each age category, per year. For each category and year, the proportion of actual users to potential users will be given in percentages, and for any year following the starting year of 2017, the relative growth of each proportion to the preceding year will also be given. As this research was not subject to the Medical Research Involving Human Subjects Act (WMO), approval from the Medical Ethical Review Board was not necessary.

## 3. Results

Table 1 shows the potential users and the actual users, their relative percentages, and the percentage growth relative to the preceding year.

Overall, the total population of potential users has stayed relatively consistent, with the exception of 2020 and 2022, in which they declined, temporarily in 2020, and potentially temporarily in 2022. In 2020, this decline occurred mostly in the 45–64 and 75–74 categories, in 2022 mostly in the 0–24, 25–44 and 45–64 categories, whereas 65–74 y.o. saw an increase.

The population of is-CGM users, in general, continued to grow throughout the study period, even if the rate at which it did so slowed down towards the end. In 2017, is-CGM was only reimbursed sporadically, which was due to the retrospective nature of the reimbursement introduced in 2018 (i.e., those that were eligible for the reimbursements in 2018 and had already started using the devices prior to that, could have the prior usage also reimbursed). From 2018 onwards, after the start of reimbursements [4], prescription increased drastically, with those aged 0–24 seeing the most widespread reimbursed usage, and those aged ≥75 the least. In 2020, is-CGM usage had increased, with growth continuing over time. During this period, those aged 0–24 formed the largest population (relative to their eligible population); however, they were overtaken in 2022 by those aged 25–44. During the same period, those in older age categories continued to lag behind, with usage decreasing continuously as age increased, even if these age categories also experienced substantial growth over the years.

## 4. Discussion

We aimed to investigate the adoption of is-CGM technology in the Netherlands, illustrated by the rate of reimbursement relative to the eligible population, and any differences therein between various age categories. As shown by the data, is-CGM technology has been increasingly reimbursed to larger proportions of the eligible population, similar to earlier studies within the Netherlands [11], as well as studies performed elsewhere [15].

The two periods of largest relative growth, in 2018 and 2019, coincided with the inclusion of is-CGM into the national reimbursement schemes and the later expansion of the criteria for reimbursement (to all those using basal-bolus regimens for diabetes of any type), respectively. It is known that costs can be a major barrier to the adoption of healthcare technologies [5], so any measures that can lessen the financial burden for the user will most likely increase adoption. Although the data from this cross-sectional study obviously cannot prove causation, they do support this notion.

The analysis shows that is-CGM has greater adoption in younger people, 0–24 and 24–44, with reduced usage in older age categories, with those aged ≥75 using is-CGM technology slightly over half as much as those aged 0–24 years in 2022. This analysis does not include real time-CGM (rt-CGM) reimbursements, because, due to more restrictive reimbursement criteria, the eligible population cannot be identified from the GIP database. However, it is unlikely that rt-CGM alone would explain the difference in is-CGM technology between the age categories, least of which because rt-CGM technology is more accessible to children than it is to adults. The relative growth rates are larger for the elderly population, indicating that they might be catching up. This is, undoubtedly, a positive development, as the technology has much to offer to these patients, such as mitigating hypoglycemia risk and improved glycemic control and quality of life [7,9,16]. However, there is still a considerable fraction of the older population that does not use is-CGM devices, and as such, efforts must be made to maintain the increased adoption rate of the devices in this population.

The overall pattern found, that of increasing sensor usage among the elderly, which yet lags behind the younger generations, matches with studies performed abroad. A study performed in Germany found that CGM usage had increased over the years in the elderly population, yet they also found that usage decreased as age increased in both type 1 and type 2 diabetes [17]. Studies performed in the United States of America likewise found CGM users to be generally younger than non-users [18,19].

The question then becomes the following: where does this disparity in is-CGM utilization stem from? Although the present data are unable to answer this question, the previous literature suggests the following reasons: First, one such barrier could be the perception that visual impairments and decreased dexterity might obstruct the proper usage of is-CGM technology [6]. However, if the sensor is paired with the standard reader, utilization of the sensor is comparable in difficulty to that of current-day capillary blood glucose measurement tools. Note that these barriers apply even less to the age category 45–64, especially towards the younger end of the category. Furthermore, in assisted-living environments and other similar situations, the sensors could be placed by staff, potentially reducing workload burden in situations where staff would otherwise have had to perform capillary measurements [20]. Another barrier could be data overload and/or alert fatigue. This could be addressed by proper mentoring and supporting the elderly when initializing the device, tailoring the settings of the device to the patients’ needs. Lastly, the presence of both problems might be a presumption on the part of the patient and physician and should not preclude from trialing the device. If the barriers do prove insurmountable, at least the attempt has been made, and one can switch back to their preferred method of measuring. Studies employing focus-group interviews could assist in identifying the prevalence of these barriers within this population, as well as guide healthcare professionals and policy-makers on which measures could be most effective in addressing these barriers. In addition, this information could also be used by device manufacturers to further improve the usability of these devices by this population via improving the design.

Another potential explanation could be that CGM was seen as more important for those with T1DM, who are predominantly younger. Despite reimbursement being available, clinicians could be reluctant to prescribe sensors to people with type 2 diabetes (T2DM), who are likely to be older. Moreover, T1DM is typically treated in specialist centers, where clinicians may be more familiar with starting CGM-guide treatment, while many older people with T2DM would be treated in primary care settings, where clinicians might not be as familiar.

In including the is-CGM devices in the national reimbursement schemes, removal of financial barriers to its adoption has been ensured. This is evidenced by the substantial increase in is-CGM usage in the eligible population. However, financial barriers are not the only barriers to exist and must not be overlooked if equitable quality of care is to be achieved. Cross-sectional studies such as this one could assist in identifying any populations, such as the elderly, which might still experience barriers to is-CGM usage beyond the now-reduced financial ones. Potential populations that might suffer from reduced access to healthcare technologies such as is-CGM devices are ethnic minorities and those of lower socio-economic status, as has been found by studies performed abroad [5].

A strength of this study is the relative completeness of the database, as the database covers nearly 95% of the total Dutch population. However, a limitation is the reliance on reported data, which might include erroneous reports and thus misidentification of potential and actual users. This is potentially related the drop in potential users in 2020 and 2022. With reimbursement being limited to only those using a basal-bolus scheme, it is possible that a number of people with T2DM formerly regulated with basal insulin only were escalated to a basal-bolus scheme in order to receive the Freestyle Libre. These prescriptions might have then been stopped in the years following, causing the decline in potential users, while Freestyle Libre prescription might not have, potentially skewing the results. In addition, the recent advances in GLP-1 agonists and associated medications might have allowed for certain individuals with type 2 diabetes to achieve insulin independence, further lowering the number of potential users identified. Furthermore, the granularity of the available data is insufficient for any further and finer analysis and does not allow for the identification of any specific ages beyond which is-CGM utilization might drop precipitously. Additionally, as this study was performed solely on reimbursement data, we had no access to clinical data, and as such, cannot ascertain any effects as to diabetes type, HbA1c, and other glycemic parameters, as well as treatment satisfaction and any interactions with age that those factors might have. However, based on other cohort studies in countries with similar populations to the Netherlands, we estimate the proportion of insulin-dependent type 2 diabetes to be 10–20% of all type 2 diabetes [21]. Considering that approximately 10% of all diabetes cases in the Netherlands are type 1 diabetes, which is treated with insulin by definition, we then estimate that, within the population of individuals with insulin-dependent diabetes, and thus the potential users in our study, approximately 36–53% have type 1 diabetes. Lastly, regarding is-CGM reimbursement data, when the subgroup sizes numbered <100, the numbers were automatically rounded to the nearest 10. This introduced small measurement errors into the analysis. However, such measurement errors were small enough to not meaningfully affect the general conclusions of this article.

## 5. Conclusions

While is-CGM technology adoption has continued to increase since its introduction, with major increases in reimbursed prescriptions, the current data derived from the Dutch population demonstrates that older age categories still lag behind, with the eldest lagging behind the most, despite larger relative growth rates in recent years. To maintain these developments, due to the potential benefits such devices offer, we argue that attempts to address this disparity must be made. One such measure would be the equal offering of the devices by addressing any preconceived notions regarding the suitability of the user for the device and by ensuring proper support and training during the starting phases of using the device [5].

## Figures and Tables

**Table 1:** Overview of the potential (those reimbursed for fast-acting insulin) and actual users (those reimbursed for is-CGM usage) of is-CGM technology, given as absolute numbers, relative percentages, and percentage growth relative to the preceding year, in total and per age category, from 2017 to 2022. Abbreviations: y.o., years old.

	Total	0–24 y.o.	25–44 y.o.	45–64 y.o.	65–74 y.o.	≥75 y.o.
2017						
Potential users (*n*)	172,799	13,474	24,645	60,027	41,578	33,075
Actual users (*n*)	38	23	7	5	3	0
Percentage (%)	0.02	0.17	0.03	0.01	0.01	0.00
2018						
Potential users (*n*)	172,958	13,617	24,747	59,134	41,591	33,869
Actual users (*n*)	1613	740	350	400	94	29
Percentage (%)	0.93	5.43	1.41	0.68	0.23	0.09
Growth relative to the preceding year (%)	4140.8	3083.7	4.879.4	8020.8	3032.4	N/A
2019						
Potential users (*n*)	171,894	13,730	25,256	57,438	40,837	34,633
Actual users (*n*)	12,692	3400	3580	4210	1120	382
Percentage	7.38	24.76	14.17	7.33	2.74	1.10
Growth relative to the preceding year (%)	691.7	355.8	902.2	983.6	1113.5	1188.2
2020						
Potential users (*n*)	168,021	13,344	25,537	55,028	39,482	34,630
Actual users (*n*)	46,370	5550	9790	17,840	8950	4240
Percentage (%)	27.60	41.59	38.34	32.42	22.67	12.24
Growth relative to the preceding year (%)	273.8	68.0	170.5	342.3	726.5	1010.0
2021						
Potential users (*n*)	174,372	13,818	27,048	56,579	39,997	36,930
Actual users (*n*)	54,940	5610	10,460	20,540	11,330	7000
Percentage (%)	31.51	40.60	38.67	36.30	28.33	18.95
Growth relative to the preceding year (%)	14.2	−2.4	0.9	12.0	25.0	54.8
2022						
Potential users (*n*)	167,515	10,721	18,214	55,150	46,525	36,905
Actual users (*n*)	82,050	6690	15,380	30,100	17,660	12,220
Percentage (%)	48.98	62.40	84.44	54.58	37.96	33.11
Relative growth to preceding year (%)	55.5	53.7	118.4	50.3	34.0	74.7

## Data Availability

The original data presented in the study are openly available in the GIP-database.
